# Distal Lung Inflammation Assessed by Alveolar Concentration of Nitric Oxide Is an Individualised Biomarker of Severe COVID-19 Pneumonia

**DOI:** 10.3390/jpm12101631

**Published:** 2022-10-02

**Authors:** Thông Hua-Huy, Sven Günther, Christine Lorut, Marielle Subileau, Frédérique Aubourg, Caroline Morbieu, Jonathan Marey, Joëlle Texereau, Isabelle Fajac, Luc Mouthon, Nicolas Roche, Anh Tuan Dinh-Xuan

**Affiliations:** 1 Lung Function & Respiratory Physiology Units, Department of Respiratory Physiology and Sleep Medicine, Cochin & George Pompidou Hospitals, Assistance Publique–Hôpitaux de Paris (APHP) Centre, University Paris Cité, 75014 Paris, France; 2 Department of Pulmonary Medicine, Cochin Hospital, APHP Centre, Institut Cochin (UMR 1016), University Paris Cité, 75014 Paris, France; 3 Department of Internal Medicine, Cochin Hospital, APHP Centre, University Paris Cité, 75014 Paris, France

**Keywords:** exhaled nitric oxide, alveolar nitric oxide, FeNO, CaNO, COVID-19, SARS-CoV-2, inflammation, pneumonia

## Abstract

Pulmonary sequelae as assessed by pulmonary function tests (PFTs) are often reported in patients infected by SARS-CoV-2 during the post-COVID-19 period. Little is known, however, about the status of pulmonary inflammation during clinical recovery after patients’ discharge from the hospitals. We prospectively measured PFTs coupled with the exhaled nitric oxide (NO) stemming from the proximal airways (FeNO) and the distal lung (CaNO) in 169 consecutive patients with varying degrees of the severity of COVID-19 six weeks to one year after acute infection by SARS-CoV-2. The proportions of patients with abnormal PFTs, defined as the presence of either obstructive/restrictive patterns or impaired lung gas transfer, or both, increased with the severity of the initial lung disease (15, 30, and 52% in patients with mild, moderate, and severe COVID-19). FeNO values remained within normal ranges and did not differ between the three groups of patients. CaNO, however, was significantly higher in patients with severe or critical COVID-19, compared with patients with milder forms of the disease. There was also an inverse relationship between CaNO and DLCO. We conclude that the residual inflammation of the distal lung is still present in the post-COVID-19 follow-up period, in particular, in those patients with an initially severe form of COVID-19. This long-lasting alveolar inflammation might contribute to the long-term development of pulmonary fibrosis and warrants the regular monitoring of exhaled NO together with PFTs in patients with COVID-19.

## 1. Introduction

Almost three years after its outbreak, the pandemic related to the severe acute respiratory syndrome coronavirus 2 (SARS-CoV-2) and coronavirus disease 2019 (COVID-19) is responsible for more than 6 million deaths worldwide [1] and continues to affect our daily lives. Due to its airborne nature, SARS-CoV-2 readily infects the respiratory tract after the binding of the viral spike protein to the angiotensin-converting enzyme 2 (ACE-2), a ubiquitous membrane receptor abundantly expressed on various lung cells. Whilst most individuals infected by SARS-CoV-2 remain symptom-free, others eventually develop pulmonary pneumoniae, which in turn profoundly impairs pulmonary gas exchange, leading to hospitalisation in intensive care units or even death [2]. As with all acute lung injuries, questions related to the long-term sequelae of lung function rapidly arose for all COVID-19 patients, irrespective of the initial severity of the disease [3]. Among these sequelae, pulmonary fibrosis is considered one of the most feared complications of COVID-19. Lung fibrotic lesions insidiously spread when dysregulated alveolar tissue repair mechanisms take over normal response to the inflammation of the distal lung after viral cell infection and tissue invasion [4].

Although many studies measuring pulmonary function tests (PFTs) have either reported the existence of transient pulmonary obstructive or restrictive patterns [5,6] and concurred on the high frequency of lung gas exchange impairment [3], only a few have monitored airway inflammation in COVID-19 survivors [7,8]. Among all the available biomarkers that are readily available, the noninvasive measurement of exhaled nitric oxide (NO) is commonly used to assess the inflammation of the lungs in patients with various respiratory diseases [9]. For example, the inflammation of the proximal airways can be monitored in asthmatic patients using the fractional concentration of NO measured with an expiratory flow of 50 mL·s^−1^ (FeNO) [10,11] whilst the inflammation of the distal lung, i.e., the small airways and/or the alveoli, can be assessed with the calculation of the alveolar concentration of NO (CaNO) [12,13].

As the site of inflammation in the airways does matter in the subsequent evolution of pulmonary involvement, we prospectively assessed the inflammation of the proximal airways and in the distal lung of patients infected by SARS-CoV-2 by measuring FeNO and CaNO, respectively, together with full PFTs in COVID-19 survivors with mild, moderate, and severe pulmonary pneumonia. The aim of our study was two-fold. First, we wanted to see whether FeNO and CaNO differ between COVID-19 survivors with mild, moderate, and severe pulmonary pneumonia. Secondly, if such differences exist, we would then study the possible links between residual inflammation and PFT impairment in those COVID-19 patients depending on their initial degree of the severity of pulmonary pneumonia. We believe that answering these questions will help to better understand the underlying mechanisms leading to the post-acute sequelae of COVID-19 (PASC), or long COVID [14].

## 2. Materials and Methods

### 2.1. Subjects

The inclusion period spanned from 10 May 2020 to 9 April 2021. All the patients were followed up at either Department of Pulmonary Medicine or the Department of Internal Medicine at Cochin University Hospital, Paris, France. In those patients with previously resolved SARS-CoV-2 infection (≥6 weeks from the onset of respiratory symptoms related to COVID-19) and in a clinically stable condition, on the same day, we performed nitric oxide (NO) measurements in the exhaled air and pulmonary function tests (PFTs), including the diffusion capacity of the lung for carbon monoxide (DLCO), as part of our routine post-COVID-19 follow-up protocol [15]. The patients with the presence of recent upper airway infection or pneumonia (<1 month), medical history of chronic respiratory diseases modifying the lung production of NO such as high-type 2 severe asthma, systemic sclerosis (SSc)-related interstitial lung disease (ILD), and severe obstructive sleep apnoea syndrome were excluded [12,16]. All lung functional tests and exhaled nitric oxide measurement have been routinely performed in our patients with informed consent agreement. The study complied with our institutional rules and therefore waived the need for specific consent, as judged by our local ethics committee. 

The patients were divided into three groups according to the severity of their acute SARS-CoV-2 infection. Mild disease consisted of outpatients with or without nasopharyngeal symptoms, no need for oxygen supply, and no sign of pneumonia. The patients in the moderate disease group had pneumonia evidenced by chest high-resolution computed tomography (HRCT) with oxygen supply up to 15 L·min^−1^. The severe/critical disease patients’ group included those patients who were hospitalised in the Intensive Care Unit (ICU), treated by either high-flow oxygen with a nasal cannula and/or mechanical ventilation for more than 24 h [17]. Pulmonary involvement during acute SARS-CoV-2 infection was retrieved, and the patients were divided into 4 groups according to the extent of their initial lung injury as assessed by lung HRCT: mild (<10%), moderate (10 to 25%), and extended/severe (>25%).

### 2.2. Exhaled Nitric Oxide Measurement

The exhaled nitric oxide (exNO) was measured using an electrochemical device (FeNO^+^ Hyp’air, MGC Diagnostics Corporation, B-5503 Sorinnes, Belgium) according to standard recommendations from the American Thoracic Society/European Respiratory Society (ATS/ERS) [18]. All the patients performed exNO measurements prior to any PFTs including a 6 min walk test, as hyperventilation could modify the exNO concentration. Briefly, after full inspiration to the total lung capacity (TLC) with a NO-free air, the patient exhaled against a device, yielding a positive resistance that was continuously kept between 5 cmH_2_O and 20 cmH_2_O to generate stable expiratory flow rates of 50, 100, and 150 mL/s. For each expiratory flow rate level, at least two measurements with reproducible results (variation coefficient less than 10%) were made. A third measurement was added if necessary to fulfil the reproducibility requirement. The mean values of NO production at each expiratory flow rate were used to determine flow-independent parameters. The alveolar concentration of exhaled nitric oxide (CaNO) and the maximal conducting airway flux of NO (J’awNO) were calculated using the two-compartment model, as previously described [19,20].

### 2.3. Lung Function Measurement

PFTs were performed (MasterScreen Body, CareFusion, Höchberg, Germany) following the American Thoracic Society/European Respiratory Society recommendations [21]. The Global Lung Initiative (GLI) reference values were applied for spirometry (the forced vital capacity (FVC) and the forced expiratory volume in one second (FEV1)) [22], lung volumes (the total lung capacity (TLC), the functional residual capacity (FRC), and the residual volume (RV)) [23], and the diffusion capacity of the lung for carbon monoxide (DLCO), the alveolar volume (AV), and the carbon monoxide transfer coefficient (KCO) [24]. An obstructive ventilator pattern was defined by a reduced FEV1/FVC ratio below the 5th percentile of the predicted value, whereas a restrictive ventilator pattern was characterised by a decrease in the TLC below the 5th percentile of the predicted value [21].

### 2.4. Statistical Analyses

The results are presented as mean and standard deviation, or number (percentage), as appropriate. The comparisons between COVID-19 patients with mild, moderate, and severe/critical diseases were performed using an ANOVA test coupled with Tukey’s post hoc test for continuous variables, and a chi-squared or Fischer’s exact test for categorical ones. The correlations between the main parameters of PFTs (including FVC, FEV_1_, TLC, FRC, RV, DLCO, AV, and KCO) and exhaled NO (CaNO and FeNO) were performed using Pearson’s method. A *p*-value equal to or less than 0.05 was considered statistically significant. Statistical analyses were performed using IBM SPSS Statistics 20.0 (Chicago, IL, USA). 

## 3. Results

### 3.1. Study Cohort

**Figure 1:**
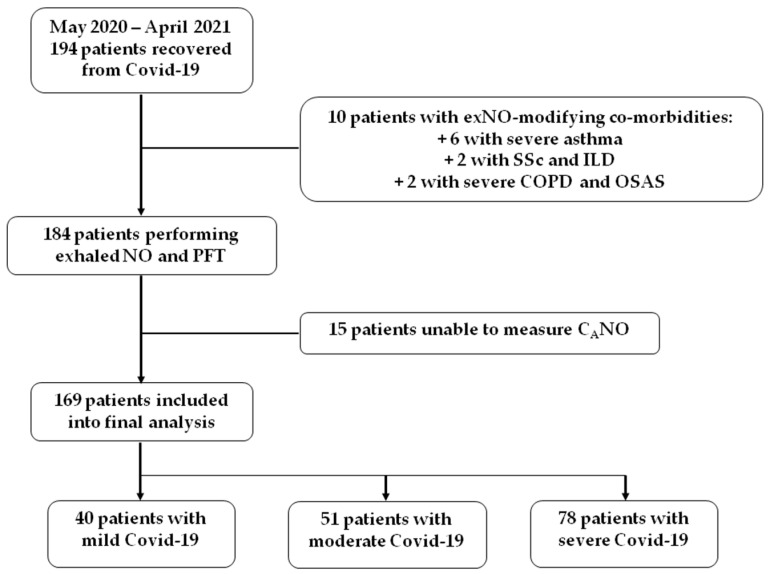
Patient recruitment, study, and final analysis. The patients were enrolled between 10 May 2020 and 9 April 2021 to perform the exhaled nitric oxide (exNO) and pulmonary function tests (PFTs) during routine surveillance after SARS-CoV-2 infection (COVID-19). SSc: systemic sclerosis; ILD: interstitial lung disease; COPD: chronic obstructive pulmonary disease; OSAS: obstructive sleep apnoea syndrome; CaNO: alveolar concentration of the exhaled nitric oxide.

Between 10 May 2020 and 9 April 2021, 194 patients who recovered from SARS-CoV-2 infection (COVID-19) were addressed to our Department of Respiratory Physiology for routine follow-up and PFT measurement. Ten patients with a history of chronic respiratory diseases that might affect NO production by the lung were excluded from the cohort. For the exNO measurement, 5 patients unable to perform high flow rate expiration (for CaNO calculation) and 10 others unable to perform any constant flow rate expiration (for FeNO and CaNO) were also excluded from the final analysis. The results from 169 patients with valid exNO measurements were finally kept for analysis according to the severity of their initial COVID-19 infection, as previously described in Materials and Methods (Figure 1).

### 3.2. Characteristics of the Studied Population

The patients from the moderate and severe disease groups were older than those with mild disease (58.7 ± 12.7 years and 60.7 ± 11.7 years versus 48.7 ± 14.3 years, respectively, *p* < 0.001). There were more men (than women) in the moderate and severe disease groups, compared with those in the mild disease group (*p* < 0.001) (Table 1). The body mass index (BMI) and obesity frequency did not differ between the three groups of patients (*p* > 0.05). There were more patients with a history of systemic hypertension and diabetes in the moderate and severe disease groups than those in the mild disease group (*p* = 0.045 and *p* = 0.004, respectively). The length of the time from the onset of the respiratory symptoms related to COVID-19 to exNO measurements varied from 42 to 364 days and did not differ between the three groups (Table 1).

Lung injury, as evidenced by the initial HRCT, was mild (72.5%) to moderate (27.5%) in patients with mild disease. Severe/extended (>25%) lung damage was found in the severe/critical disease group in 85% of the patients. Regarding the care modalities, most patients with mild disease were kept at home (97.5%) while the patients with a moderate form of the disease were hospitalised in traditional care units (84%) and severe/critical disease patients were admitted to intensive care units (85%) (Table 1).

### 3.3. Pulmonary Function Tests and Exhaled Nitric Oxide Measurement

Abnormal lung function was found in many patients 5 months after SARS-CoV-2 infection. In particular, the patients with severe disease had a significant decrease in DLCO, TLC, and FVC, compared with the patients with mild disease (Table 2). An abnormal PFT was defined as the presence of restrictive or obstructive patterns or decreased DLCO under the lower limit of normal (GLI norm). An abnormal PFT was more frequent in the patients belonging to either the severe/critical or moderate disease groups (67% and 59%, respectively), compared with patients with mild disease (37.5%, *p* = 0.005) (Table 2).

**Figure 2:**
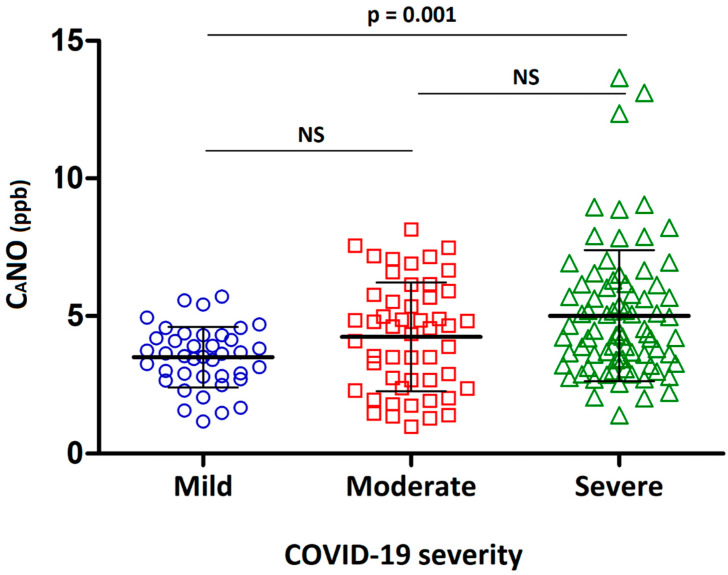
Alveolar concentration of exhaled nitric oxide (CaNO) in patients with recovered SARS-CoV-2 pneumonia.COVID-19 severity was determined as described previously in Materials and Methods (mild disease group with 40 patients; moderate 51 patients; severe 78 patients). For each group, the central thickened black bar presents arithmetical mean of CaNO (ppb) of the group, and two black thin bars present the standard derivations (SDs). Comparison between the three groups was performed using ANOVA analysis coupled with Tukey’s post hoc tests. NS: no statistically significant (*p* > 0.05). This figure was drawn using GraphPad Prism version 5.01(GraphPad Software, San Diego, CA, USA) for Windows.

Measuring the exhaled NO concentrations using the two-compartment model method generated three flow-independent parameters, namely FeNO, J’awNO, and CaNO. While the first two parameters reflect the NO derived from the proximal airways, CaNO is thought to represent the NO produced in the distal lung including the bronchioloalveolar spaces [19]. No statistical difference was found regarding FeNO and J’awNO between the three groups of patients with mild, moderate, and severe COVID-19-related lung disease. CaNO was, however, significantly increased (*p* = 0.001) in the severe/critical disease group (5.0 ± 2.4 ppb), compared with the mild disease group (3.5 ± 1.1 ppb) (Table 2 and Figure 2).

The severity of SARS-CoV-2 pneumonia can also be determined by the extent of lung parenchymal involvement quantified by the initial thoracic HRCT and/or the care modalities required during an acute period (home care, traditional hospitalisation, or intensive care unit). Among the three exhaled NO parameters, only CaNO was significantly increased in the most severe COVID-19 group, compared with other groups (Table 3: thoracic HRCT extent; *p* = 0.003; and Table 4: care modalities; *p* = 0.001).

**Figure 3:**
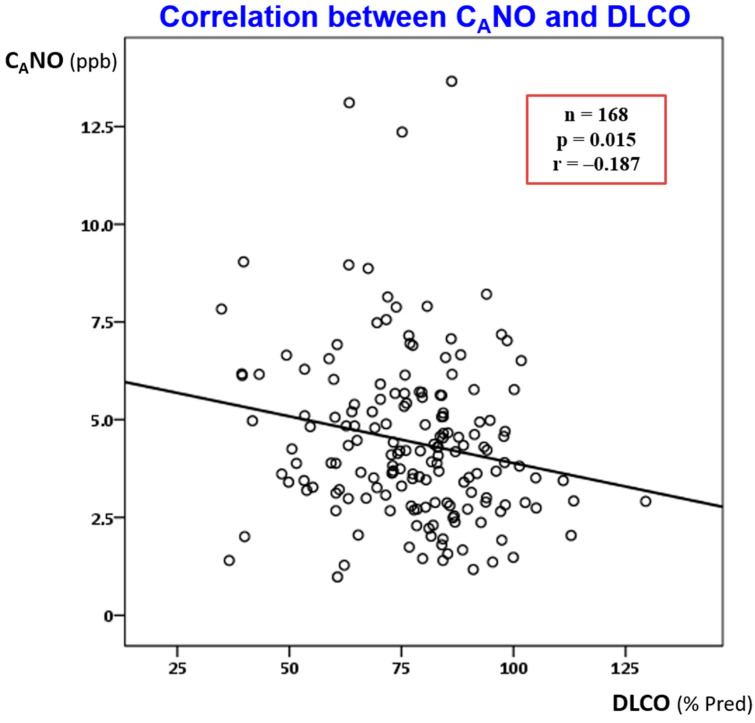
Correlations between alveolar concentration of exhaled nitric oxide (CaNO) and diffusion capacity of the lung for carbon monoxide (DLCO) in patients with recovered SARS-CoV-2 pneumonia.

Finally, we investigated the possible correlation between the main parameters of PFTs (including FVC, FEV_1_, TLC, FRC, RV, DLCO, AV, and KCO) and exhaled NO, CaNO and FeNO. Only a weak inverse correlation (rho = −0.187) between CaNO and DLCO reached the statistically significant threshold (*p* = 0.015), as presented in Figure 3.

The correlation between the alveolar concentration of exhaled nitric oxide (CaNO) and the diffusion capacity of the lung for carbon monoxide (DLCO) in patients with recovered SARS-CoV-2 pneumonia was investigated using Pearson’s method, with *p* < 0.05 and a negative Pearson coefficient of correlation −0.187.

## 4. Discussion

In this prospective study conducted on 169 consecutive COVID-19 patients with varying degrees of severity, we found that the proportions of patients with abnormal PFTs increased with the severity of initial lung diseases. Pulmonary inflammation measured at different levels of the airways was not uniformly present. FeNO values, mirroring the inflammation in the proximal airways, remained within normal ranges and did not differ between the three groups of patients. CaNO, characterising the inflammation in the distal lung, was, however, significantly higher in patients with severe or critical COVID-19, compared with patients with milder forms of the disease.

Among the numerous long-lasting effects of COVID-19, known as PASC [25], which might affect many organs, pulmonary sequelae are by far the most frequently reported to date [3]. PFT measurements identified three main features of lung function impairment, namely obstructive pattern, restrictive pattern, and lung gas exchange disturbances in those COVID-19 survivors without any known pulmonary disorders prior to their infection by SARS-CoV-2 [3,5,6]. The ratio of the forced expiratory volume in one second (FEV_1_) to the forced vital capacity (FVC) was used to define the bronchial obstructive pattern [3,5,6]. A restrictive pattern was sought by looking at either a reduction in the total lung capacity (TLC) or the combination of low FVC and high FEV_1_/FVC ratio when the TLC could not be measured [3]. Lung gas exchange was assessed by the single breath carbon monoxide lung diffusing capacity (DLCO) measurements [3,5,6]. One feature constantly observed in all studies was the high prevalence of reduced DLCO, the medium-to-high prevalence of restrictive patterns, and the relatively low prevalence of obstructive patterns [3,5,6] in those COVID-19 patients in whom PFTs were assessed one to twelve months after their initial acute infection. The results of our study are consistent with the previous data reported in the literature with only 2% (4 out of 169) of patients with COVID-19 having a reduced FEV_1_/FVC ratio consistent with an obstructive pattern (Table 2). The restrictive pattern was more frequently diagnosed in 18% (31 out of 169) of COVID-19 patients with two-thirds (20 out of 31) of the patients belonging to the severe or critical group (Table 2). As previously reported, reduced DLCO was the most frequent abnormal result in PFTs, being present in 40% (68 out of 169) of patients with COVID-19 among whom 60% (41 out of 68) of the patients were the most severely affected during the acute infection phase (Table 2).

Many mechanisms might account for impaired lung gas transfer in patients with COVID-19 [26]. DLCO can be markedly reduced during the initial phase of acute lung injury as a consequence of inflammatory processes affecting the vessel–lung tissue interface, thereby impairing oxygen transfer across the alveolar–capillary membrane in both the acute respiratory distress syndrome (ARDS) related to common causes [27] or resulting from SARS-CoV-2 infection [28]. Impaired DLCO also belongs to the long-term complications observed in the survivors of severe ARDS [29] and reflects here the occurrence of irreversible lung fibrotic lesions following COVID-19 [30,31]. Recent evidence suggests that SARS-CoV-2 may induce lung fibrogenesis through different biological pathways, but the clinical relevance of these processes is still debated [32]. Irrespective of the nature of the underlying biological pathways leading to pulmonary fibrosis, one feature, i.e., the essential role of inflammation as an important trigger for tissue fibrosis, seems to reach a consensus among the scientific community [33].

Assessing alveolar inflammation has not always been an easy task. The historical approach implies invasive processes such as bronchoalveolar lavage (BAL) coupled with the measurement of inflammatory biomarkers in the BAL fluid. The recent development of NO quantification in the exhaled air now provides a noninvasive tool yielding reproducible and reliable results of this universal gaseous biomarker of inflammation [10]. Using lung modeling and the measurements of NO production at different expiratory flow rates, it is now possible to distinguish inflammation in the proximal airways (reflected by FeNO) from that occurring in the distal lung (assessed by CaNO values) [13].

The results from our study related to the measurements of NO in the exhaled air from patients with COVID-19 can be interpreted as follows: First, bronchial T2 or T2-like inflammation with high values of FeNO could not be observed in any of the three groups of COVID-19 patients, as the mean FeNO was below 25 ppb in patients with mild, moderate, and severe COVID-19 six weeks to one year after the acute infection (Table 2). Secondly, distal bronchioloalveolar inflammation reflected by CaNO was significantly greater in patients with severe COVID-19, compared with patients having a milder form of the disease (Table 2 and Figure 2).

Our results are consistent with recently published studies finding that FeNO values did not differ between COVID-19 patients with varying degrees of severity [34,35,36,37]. These observations have prompted some investigators to cast doubt on the need of measuring exhaled NO as a biomarker in post-COVID-19 patient monitoring [36,37]. As already discussed, a key feature related to the physiological meanings of different NO parameters must, however, constantly be emphasised [13]. Many already published studies only investigated FeNO [33,34,35,36,37], a biomarker of proximal bronchial inflammation [10], that is unlikely to occur 6 to 52 weeks after the acute phase in those COVID-19 patients without a medical history of asthma. That FeNO remained within normal ranges and did not differ between different groups of patients, as observed in our study and many others [7,8,33,34,35,36,37], is also consistent with the relatively low percentage of patients with bronchial obstructive patterns during COVID-19 follow-up studies [3,5,6], and the hypothesis that bronchial obstructive lung disease is an unlikely feature of PASC [3]. Of greater concern, however, is the risk of pulmonary fibrosis that might occur as a long-term complication of COVID-19 even in those patients free of ILD prior to infection by SARS-CoV-2 [30,32]. Although reduced DLCO is a reliable and sensitive marker of impaired lung gas transfer [38,39], it does not necessarily herald the occurrence of subsequent irreversible pulmonary fibrosis, as many patients with COVID-19 eventually recovered and regained normal lung function 6 to 12 months after initial infection [3]. Unfortunately, pulmonary fibrosis as a long-term sequela of COVID-19 will occur in some patients, with a prevalence that is yet unknown, and for whom no predictive biomarker is available to date.

There are theoretical grounds to hypothesise that prolonged inflammation in the distal lung favours the likelihood of tissue remodelling and the development of subsequent fibrotic lesions, hence increasing the risk of irreversible lung fibrosis in post-COVID periods. Our findings of increased CaNO in the most severely affected patients with COVID-19, compared with those with milder disease (Table 2 and Figure 2), and an inverse relationship between CaNO and DLCO values (Figure 3) have several implications. First, the inflammation in the distal lung is a marker of COVID-19 severity in the initial phase, in the same way as a pulmonary restrictive pattern and impaired DLCO [3,5,6]. Secondly, the weak but significant inverse relationship between CaNO and DLCO suggests possible links between the ongoing inflammation of the distal lung and reduced lung gas transfer. We do not know whether these two parameters, alone or in combination, will reliably predict the occurrence of subsequent pulmonary fibrosis. The answer to this question can only be provided after a long-term follow-up study of large cohorts of patients with COVID-19 patients.

Previous works published by our centre revealed that measuring CaNO not only helps to detect ILD [40] but also facilitates the assessment of the severity of lung disease [41] and the prediction of lung function deterioration [20] in patients with SSc. Whether monitoring the inflammation of the distal lung will be similarly useful in COVID-19 patients is still to be unequivocally shown. The results from our study and from the one recently published by Cameli et al. [42] allow us to confidently suggest additional measurements of CaNO together with PFTs in all patients with impaired lung function during post-COVID-19 long-term follow-up. Further studies will help our understanding of whether and how this measurement can add to standard lung function tests to help predict long-term outcomes and eventually contribute to guiding the management of this disease.

## Figures and Tables

**Table 1:** Characteristics of the studied population and SARS-CoV-2 pneumonia.

	Mild Disease	Moderate	Severe/Critical	*p*-Value
	N = 40	N = 51	N = 78	(ANOVA)
Age; years	48.7 ± 14.3	58.7 ± 12.7 ^##^	60.7 ± 11.7 ^###^	<0.001
Male; n (%)	14 (35)	38 (74.5)	58 (74)	<0.001
Height; cm	167 ± 8	173 ± 7	170 ± 8 ^##^	0.011
Weight; kg	73 ± 17	84 ± 15 ^##^	79 ± 14	0.002
BMI; kg·m^−2^	25.8 ± 5.3	28.3 ± 4.4	27.4 ± 4.9	0.058
Obesity; n (%)	9 (22.5)	16 (31)	15 (19)	0.279
Smoker				
▪ Nonsmoker	31 (77.5)	34 (67)	53 (68)	
▪ Active; n (%)	4 (10)	3 (6)	3 (4)	0.274
▪ Former; n (%)	5 (12.5)	14 (27)	22 (28)	
Co-morbidities				
▪ Lung disease	10 (25)	12 (24)	12 (15)	0.359
▪ Heart disease	2 (5)	6 (12)	12 (15)	0.255
▪ Hypertension	6 (15)	16 (31)	29 (37)	0.045
▪ Diabetes	2 (5)	9 (18)	24 (31)	0.004
▪ Cancer	1 (2.5)	4 (8)	6 (8)	0.5
Time from COVID-19; day	142 ± 41	141 ± 52	135 ± 48	0.712
First thoracic HRCT severity				
No/Mild injury (<10%)	29 (72.5)	0 (0)	0 (0)	
Moderate injury (10–25%)	11 (27.5)	28 (55)	12 (15)	<0.001
Severe/extended injury (>25%)	0 (0)	23 (45)	66 (85)	
Care modality				
Outpatient	39 (97.5)	2 (4)	0 (0)	
Traditional hospitalisation	1 (2.5)	43 (84)	12 (15)	<0.001
ICU admission	0 (0)	6 (12)	66 (85)	

Results are presented as mean and standard deviation, or number (percentage), as appropriate. Comparisons between patients with mild, moderate, and severe/critical COVID-19 were performed using ANOVA test coupled with Tukey’s post hoc test for continuous variables, and chi-squared or Fischer’s exact test for categorical ones. ^##^: *p* < 0.01, ^###^: *p* < 0.001, compared with mild disease group. BMI: body mass index; HRCT: high-resolution computed tomography; ICU: intensive care unit.

**Table 2:** Pulmonary function tests and exhaled nitric oxide measurement in patients with recovered SARS-CoV-2 pneumonia.

	Mild Disease	Moderate	Severe/Critical	*p*-Value
	N = 40	N = 51	N = 78	(ANOVA)
Spirometry				
▪ FVC; % pred	92 ± 16	89 ± 17	84 ± 16 ^#^	0.035
▪ FEV_1_; % pred	91 ± 14	90 ± 16	88 ± 18	0.666
▪ FEV_1_/FVC; %	0.81 ± 0.07	0.79 ± 0.07	0.82 ± 0.08 ^#^	0.044
Obstructive pattern (FEV_1_/FVC < LLN-GLI)	1 (2.5)	2 (4)	1 (1)	0.627
Lung volumes				
▪ TLC; % pred	104 ± 14	95 ± 18 ^#^	89 ± 14 ^###^	< 0.001
▪ RV; % pred	111 ± 22	96 ± 18 ^## §§^	84 ± 20 ^###^	< 0.001
▪ FRC; % pred	102 ± 23	95 ± 23	88 ± 19 ^##^	0.003
Restrictive pattern (TLC < LLN-GLI)	2 (5)	8 (16)	21 (27)	0.012
Lung diffusion capacity (µ)				
▪ DLCO; % pred	88 ± 13	79 ± 12 ^# §^	71 ± 17 ^###^	< 0.001
▪ KCO; % pred	92 ± 13	92 ± 16	91 ± 40	0.989
▪ AV; % pred	95 ± 14	87 ± 15 ^##^	81 ± 13 ^###^	< 0.001
Lung diffusion impairment (DLCO < LLN-GLI)	7 (17.5)	20 (39)	41 (53)	0.001
Abnormal PFT	15 (37.5)	30 (59)	52 (67)	0.005
CaNO; ppb	3.5 ± 1.1	4.2 ± 2.0	5.0 ± 2.4 ^##^	0.001
CaNO > 5 ppb	3 (7.5)	16 (31)	34 (44)	<0.001
J’awNO; nl/mn	49.6 ± 23.6	55.8 ± 29.5	54.9 ± 29.9	0.264
FeNO; ppb	19.4 ± 7.5	21.6 ± 9.8	22.6 ± 10.8	0.541

Results are presented as mean and standard deviation, or number (percentage), as appropriate. Comparisons between three groups of patients with mild, moderate, and severe/critical COVID-19 were performed using ANOVA test coupled with Tukey’s post hoc test for continuous variables, and chi-squared or Fischer’s exact test for categorical ones. ^#^: *p* < 0.05; ^##^: *p* < 0.01; ^###^: *p* < 0.001, compared with mild disease group. ^§^: *p* < 0.05; ^§§^: *p* < 0.01, compared with severe/critical disease group. CaNO: Alveolar concentration of exhaled nitric oxide (NO); J’awNO: maximal conducting airway flux of NO; FeNO: fractional expired nitric oxide concentration at expiratory flow rate of 50 mL/s; (FeNO^+^ Hyp’air, MGC diagnostics). FVC: Forced vital capacity; FEV_1_: forced expiratory volume in one second; LLN-GLI: Lower limit of normal (LLN) according to the Global Lung Initiative (GLI), as previously described in Materials and Methods section; TLC: total lung capacity; RV: residual volume; FRC: functional residual capacity; DLCO: pulmonary diffusing capacity for carbon monoxide; KCO: pulmonary diffusing coefficient for carbon monoxide; AV: alveolar volume; PFT: pulmonary function testing. Abnormal PFT was defined as the presence of restrictive and/or obstructive patterns and/or decreased DLCO under the lower limit of norm (GLI norm). (^µ^): one patient in the group of moderate COVID-19 could not perform the DLCO measurement (n = 50 in this group for DLCO).

**Table 3:** Relation between the extent of acute lung injury on thoracic HRCT and exhaled nitric oxide in patients with recovered SARS-CoV-2 pneumonia.

	Mild	Moderate	Extended/Severe	*p*-Value (ANOVA)
Cases (n)	29	51	89	
CaNO; ppb	3.6 ± 1.1 ^##^	4.0 ± 1.7 ^#^	4.9 ± 2.4	0.003
J’awNO; nl/mn	50.1 ± 23.9	56.3 ± 23.9	53.8 ± 31.9	0.65
FeNO; ppb	19.6 ± 7.6	21.9 ± 8.3	22.0 ± 11.2	0.5

Results are presented as mean and standard deviation. Comparison between the 3 groups of patients with mild (HRCT unneeded or with less than 10% lung injury), moderate (between 10% and 25% lung injury on HRCT), extended/severe (more than 25% lung injury on HRCT) lung injury was performed using ANOVA test coupled with Tukey’s post hoc test. ^#^: *p* < 0.05 (*p* = 0.034); ^##^: *p* < 0.01 (*p* = 0.007), compared with the extended/severe HRCT lung injury group. CaNO: alveolar concentration of exhaled nitric oxide; J’awNO: maximal conducting airway flux of NO; FeNO: fractional expired nitric oxide concentration at expiratory flow rate of 50 mL/s; (FeNO^+^ Hyp’air, MGC Diagnostics, Belgium). HRCT: high-resolution chest tomography; Mild: no HRCT (unneeded) or less than 10% lung injury on HRCT; Moderate: between 10% and 25% lung injury on HRCT; Extended: between 25% and 50% lung injury on HRCT; Severe: more than 50% lung injury on HRCT.

**Table 4:** Correlations between the clinical severity of the acute COVID-19 (as reflected by the care structure during the acute period and exhaled nitric oxide in patients with recovered SARS-CoV-2 pneumonia.

	Home Care	Traditional Hospital	Intensive Care Unit	*p*-Value (ANOVA)
Cases (n)	41	56	72	
CaNO; ppb	3.5 ± 1.1	4.4 ± 2.2	5.0 ± 2.3 ^###^	0.001
J’awNO; nl/mn	49.1 ± 23.7	58.9 ± 31.3	52.8 ± 28.1	0.218
FeNO; ppb	19.2 ± 7.6	22.9 ± 10.8	21.8 ± 10.1	0.18

Results are presented as mean and standard deviation. Comparisons between the 3 groups of patients with home care, traditional hospitals, and intensive care units was performed using ANOVA test coupled with Tukey’s post hoc test. ^###^: *p* < 0.001, compared with the mild HRCT lung injury group. CaNO: alveolar concentration of exhaled nitric oxide (NO); J’awNO: maximal conducting airway flux of NO; FeNO: fractional expired nitric oxide concentration at expiratory flow rate of 50 mL/s; (FeNO^+^ Hyp’air, MGC Diagnostics, Belgium).

## Data Availability

Not applicable.
